# Altered regulation of tau phosphorylation in a mouse model of down syndrome aging

**DOI:** 10.1016/j.neurobiolaging.2011.06.025

**Published:** 2012-04

**Authors:** Olivia Sheppard, Florian Plattner, Anna Rubin, Amy Slender, Jacqueline M. Linehan, Sebastian Brandner, Victor L.J. Tybulewicz, Elizabeth M.C. Fisher, Frances K. Wiseman

**Affiliations:** a University College London Institute of Neurology, London, UK; b MRC National Institute for Medical Research, London, UK

**Keywords:** Alzheimer disease, Down syndrome, Tau, DYRK1A, GSK-3β, Phosphorylation

## Abstract

Down syndrome (DS) results from trisomy of human chromosome 21 (Hsa21) and is associated with an increased risk of Alzheimer's disease (AD). Here, using the unique transchromosomic Tc1 mouse model of DS we investigate the influence of trisomy of Hsa21 on the protein tau, which is hyperphosphorylated in Alzheimer's disease. We show that in old, but not young, Tc1 mice increased phosphorylation of tau occurs at a site suggested to be targeted by the Hsa21 encoded kinase, dual-specificity tyrosine-(Y)-phosphorylation regulated kinase 1A (DYRK1A). We show that DYRK1A is upregulated in young and old Tc1 mice, but that young trisomic mice may be protected from accumulating aberrantly phosphorylated tau. We observe that the key tau kinase, glycogen synthase kinase3-β (GSK-3β) is aberrantly phosphorylated at an inhibitory site in the aged Tc1 brain which may reduce total glycogen synthase kinase3-β activity. It is possible that a similar mechanism may also occur in people with DS.

## 1 Introduction

Down syndrome (DS) is the most common cause of genetic intellectual disability and is associated with an increased risk of Alzheimer's disease (AD). Between 30% and 70% of people aged 60 years or older who have DS develop AD (Wiseman et al., 2009). The life expectancy of people who have DS has significantly increased in the last 20 years and hence so has the incidence of AD in this population (Glasson et al., 2002). The onset of AD in people who have DS is characterized by personality changes and executive dysfunction (Ball et al., 2006). The neuropathological changes associated with AD are similar in people with and without DS (Mann, 1988). These changes included the deposition of amyloid plaques composed of amyloid-beta (Aβ) peptides and neurofibrillary tangles (NFT) that are formed of hyperphosphorylated tau. Genetic variations in the tau gene, microtubule associated protein tau (*MAPT*), are a risk factor for the age of onset of AD in people with DS (Jones et al., 2008). This may promote the formation of NFT and may play a role in the pathogenesis of AD in people with DS (Flament et al., 1990).

DS is caused by trisomy of chromosome 21 on which 242 protein encoding genes are found (www.ensembl.org, release 59). Hence trisomy of some of these genes results in an increased risk of AD, as is observed in people who have DS, perhaps by promoting the aberrant phosphorylation of tau. Moreover, aberrant phosphorylation of tau has also been reported in trisomic Ts65Dn and Ts1Cje, mice that model aspects of DS (Liu et al., 2008; Shukkur et al., 2006). Amyloid precursor protein (*APP*) is encoded on human chromosome 21 (Hsa21); trisomy of this gene is likely to contribute to the onset of AD in people with DS. Pathogenic APP species, associated with AD are proposed to interact with tau to exacerbate disease associated phenotypes (Dawson et al., 2010; Roberson et al., 2007). The Tc1 mouse model that was used in this study is not functionally trisomic for APP because of a genomic rearrangement in Hsa21 that is present in all Tc1 mice (S. Gribble, Wellcome Trust, Sanger Institute, personal communication, and Supplementary Fig. S1). Therefore in this study we are able to investigate the effect of Hsa21 trisomy on tau in absence of any potential influence of APP trisomy.

A number of recent studies have suggested that trisomy of the Hsa21-encoded, proline-directed kinase DYRK1A, which is expressed in fetal and adult brain, may contribute to the aberrant phosphorylation of tau (Martí et al., 2003). People trisomic for Hsa21 express elevated levels of DYRK1A and exhibit increased DYRK1A kinase activity in their brains (Dowjat et al., 2007; Liu et al., 2008; Lockstone et al., 2007; Wegiel et al., 2008). In vitro, tau is phosphorylated at Thr212 by DYRK1A (Liu et al., 2008; Woods et al., 2001). This site is hyperphosphorylated in patients with AD, and may contribute to disease pathogenesis (Morishima-Kawashima et al., 1995). Phosphorylation at this site also primes tau for further phosphorylation at additional sites by other kinases, such as glycogen synthase kinase3-β (GSK-3β) (Woods et al., 2001). Overexpression of DYRK1A in transgenic mouse models results in elevated phosphorylation of tau at a number of sites, and RNA interference (RNAi) knockdown of DYRK1A expression results in reduced phosphorylation of tau in cultured cells (Azorsa et al., 2010; Liu et al., 2008; Ryoo et al., 2007). DYRK1A is found in the cytosol of cells and colocalization of NFT and DYRK1A has been reported (Martí et al., 2003; Wegiel et al., 2004). Thus, trisomy of *DYRK1A* may contribute to the early onset of AD in people with DS via an effect on tau phosphorylation.

The trisomy of Hsa21 encoded genes might also have an effect on the expression and activity of kinases encoded by chromosomes other than Hsa21. For example, increased abundance of cyclin-dependent kinase 5 (CDK5) has been reported in the brains of young Ts65Dn mice that model aspects of DS (Pollonini et al., 2008). Alterations in the activity of GSK-3β and CDK5 have been linked to hyperphosphorylation of tau and may contribute to the onset of AD (Noble et al., 2003). Also, decreased activity of phosphatases such as protein phosphatase 2A (PP2A), that can dephosphorylate tau, have been associated with the development of AD in people who have DS (Liang et al., 2008). Perturbations of these proteins in people with DS may contribute to the pathogenesis of AD.

To investigate the effect of trisomy of Hsa21 on the molecular mechanisms that underlie the pathogenesis of AD, we have studied the phosphorylation of tau and abundance of key regulators of tau phosphorylation in a unique mouse model of DS in both young and aged animals. The phosphorylation of tau has been previously studied in DS mouse models which are trisomic for approximately 55% or fewer Hsa21 genes. The Tc1 mouse model of DS, used in this study, contains a freely-segregating copy of Hsa21 in addition to a full complement of mouse chromosomes (O'Doherty et al., 2005) and is trisomic for more than 75% of Hsa21 protein encoding genes, including *DYRK1A* (S. Gribble, Wellcome Trust, Sanger Institute, personal communication). The Tc1 mouse model exhibits numerous phenotypes that resemble those observed in people who have DS, including deficits in long term potentiation (LTP) in the hippocampus and learning and memory problems (Alford et al., 2010; Dunlevy et al., 2010; Galante et al., 2009; Morice et al., 2008; O'Doherty et al., 2005; Reynolds et al., 2010). Here we study tau phosphorylation and associated regulators in the most genetically complete mouse model of Hsa21 trisomy used to address these issues to date.

In aged Tc1 mice we see an increase in the phosphorylation of tau at Thr212 but that there is no such change in the brains of young Tc1 mice. Our results show that the expression of DYRK1A, a proposed tau kinase, is elevated in the brains of young adult and old Tc1 mice. Thus young Tc1 mice appear to be protected from accumulating aberrantly phosphorylated tau despite having elevated levels of DYRK1A. We also observe an increase in phosphorylation of GSK-3β at Ser9 in aged but not young Tc1 mice. GSK-3β is a key contributor to the hyperphosphorylation of tau and may be important to the phosphorylation of tau in the context of Hsa21 trisomy (Cohen and Frame, 2001; Sutherland et al., 1993). Supporting the observed alteration in phosphorylation of GSK-3β at Ser9 we show that v-akt murine thymoma viral oncogene homolog (AKT) exhibits an increase in phosphorylation in the brains of aged Tc1 mice. AKT is an upstream regulator of GSK-3β Ser9 phosphorylation and the change we see has been previously correlated with increased activity of this kinase. Therefore our data suggest the novel finding that Hsa21 trisomy may alter the activity of GSK-3β in an age-dependent manner. This mechanism may also occur in people with DS.

## 2 Methods

### 2.1 Animal welfare

Mice were housed in controlled conditions in accordance with guidance issued by the Medical Research Council in Responsibility in the Use of Animals for Medical Research (1993) and all experiments were carried out under License from the UK Home Office and with Local Ethical Review panel approval.

### 2.2 DNA extraction and genotyping

DNA was extracted from tail tip (approximately 3 mm) or ear biopsy from all samples analyzed by either the hot sodium hydroxide and tris (HOTSHOT) method (Truett et al., 2000) or the proteinase K method. For the proteinase K method tissue is lysed overnight using proteinase K digestion in nuclei lysis buffer (Promega, Madison, WI, USA), plus 0.12 M ethylenediaminetetracetic acid (EDTA) at 55 °C. Proteins are precipitated from the resultant lysate by addition of protein precipitation solution (Promega). DNA is then precipitated with isopropanol and resuspended in DNase free water. Tc1 mice were genotyped using polymerase chain reaction (PCR) (Tc1-specific primers forward: 5′-GGTTTGAGGGAACACAAAGCTTAACTCCCA-3′; reverse: 5′-ACAGAGCTACAGCCTCTGACACTATGAACT-3′; control primers forward: 5′- TTACGTCCATCGTGGACAGCAT-3′; reverse: 5′-TGGGCTGGGTGTTAGTCTTAT-3′).

Tc1 mice were taken from a colony maintained by mating Tc1 females to F1(129S8 × C57BL/6) males. Presence of the human *DYRK1A* in the Tc1 mice was checked by PCR of genomic DNA using primers specific to human *DYRK1A* sequence (forward 1: 5′- ATCCTCCTCGGGAAGAAGCC-3′, reverse 1: 5′-GTGCATTGTCCTTGCGAATC-3′; forward 2: 5′-AGCCGAGGAGAGACTGAGCAG-3′; reverse 2 5′-AGCCGGCCCCATTTTCTTAAC -3′).

### 2.3 Sequencing

PCR products were purified using QIAquick PCR purification kit (Qiagen, Sussex, UK) prior to automated fluorescence sequencing using a BigDye Terminator Ready Reaction Kit (Applied Biosystems, Carlsbad, CA, USA) on a 3130XL Genetic Analyser (Applied Biosystems) according to manufacturer's protocols.

### 2.4 RNA extraction and reverse transcription-PCR

RNA was extracted from whole brains from adult Tc1 and age- and sex-matched euploid controls. Total RNA was extracted using TRIzol reagent (Invitrogen, Paisley, UK), precipitated as per manufacturer's instructions and resuspended in DNase- and RNase-free water. Amounts of RNA were equalized and complementary DNA was generated using a standard reverse-transcription protocol using random primers (Promega), Superscript II (Invitrogen), First Strand buffer (Invitrogen), and Deoxynucleotide Triphosphates (Promega). PCR using primers which amplify a product from both mouse *Dyrk1A* and human *DYKR1A* transcripts (forward: 5′- GGAGAGACTTCAGCATGCAAAC-3′; reverse: 5′-GCTGGGTCACGGAAGGTTTG-3′) and PCR using primers designed to raised a product against human but not mouse *Dyrk1a* (forward: 5′-CAAGAAAACAGCTGATGAAGG-3′; reverse 1: 5′-GCCACTGGGCGATTCTGG-3′; reverse 2: 5′-GATACGGTCATTCTAAAGGC-3′) were used. Similarly PCR primers designed to raise a product against human but not mouse *APP* (exon 9 forward 2: 5′-AGCCAAAGAGAGGCTTGAG-3′; exon 15 reverse: 5′- CGGGCATCAACAGGCTCAA-3′; exon 14 forward 1: 5′- CTCTCATGCCATCTTTGACC-3′; exon 18 reverse 5: 5′- CTGCTCAAAGAACTTGTAG-3′) were used.

### 2.5 Tissue preparation and Western blotting

For analysis of DYRK1A abundance in hippocampus and cortex, Tc1 and aged- and sex-matched wild type littermate samples were dissected under ice cold phosphate buffered saline (PBS) before homogenization in radioimmunoprecipitation assay buffer (150 mM sodium chloride, 50 mM Tris, 1% nonidet-40, 0.5% sodium deoxycholate, 0.1% sodium dodecyl sulfate (SDS) plus complete protease inhibitors (Roche Applied Science, Basel, Switzerland) by mechanical disruption using a Dounce homogenizer. For biochemical analysis of all other proteins, hippocampus and cortex of Tc1 and age- and sex-matched wild type littermate mice were dissected in freshly prepared dissection buffer (10 mM Tris, 320 mM sucrose, 2 mM EDTA, 0.025% NaN_3_, 0.2 mM phenylarsine oxide, 0.1 mM ammonium molybdate, 50 mM sodium fluoride, 2 mM sodium orthovanadate, and 10 mM sodium pyrophosphate) on ice, as described in Plattner et al. (2006). All samples were collected between hour 1 and hour 6 of the light cycle (standard 12-hour dark, 12-hour light cycle). Individual samples of hippocampus or cortex were then homogenized on ice in P2 buffer (10 mM Tris, 320 mM sucrose, 2 mM EDTA, 0.025% NaN_3_, 0.4 mM phenylarsine oxide, 0.2 mM ammonium molybdate, 100 mM sodium fluoride, 4 mM sodium orthovanadate, and 20 mM sodium pyrophosphate) plus complete protease inhibitors (Roche Applied Science) by mechanical disruption using a Dounce homogenizer. Total protein content was determined by DC Protein Assay (radioimmunoprecipitation assay buffer samples) or Bradford assay (P2 buffer samples) (Bio-Rad, Hemel Hempstead, UK). Samples from individual animals were run separately and were not pooled.

Equal amount of total brain proteins were then denatured in SDS denaturing buffer (Invitrogen) and β-mercaptoethanol for 10 minutes at 100 °C, prior to separation by SDS-polyacrylamide gel electrophoresis using precast 4%–20% Tris-glycine gels (Invitrogen). Proteins were transferred to nitrocellulose membrane prior to blocking in 5% milk tris buffered saline (TBS) (50 mM Tris, 150 mM NaCl, pH 7.6) for 1 hour before incubating overnight with primary antibody diluted in 1% bovine serum albumin (BSA)/TBS at 4 °C. After washing in TBST (50 mM Tris, 150 mM NaCl, 0.05% Tween 20, pH 7.6) membranes were incubated with an infrared-dye (800 and 680 CW) conjugated goat, anti-mouse and anti-rabbit, secondary antibodies (Li-Cor Odyssey, Lincoln, NE, USA) for 1 hour in the dark, prior to imaging using an Odyssey Infrared Imaging System. Signal (integrated intensity) was measured from manually assigned bands. See-Blue plus 2 (Invitrogen) or Odyssey Protein molecular weight markers (Li-Cor Odyssey) was used as a molecular weight marker. For total tau, total AKT, and total GSK-3β measurements nitrocellulose membranes previously incubated with PHF1, AT8, Tau-threonine212, GSK-3β phospho-serine9, GSK-3β phosphor-tyrosine216, and AKT phosphor-serine473 were stripped of primary and secondary antibody signal by washing for 30 minutes in stripping solution (2% SDS, 0.007% β-mercaptoethanol, phosphate-buffered saline), prior to blocking in 5% milk TBS and incubation with the new primary antibody. Similarly blots probed with antibodies against DYRK1A, protein phosphatase (PP1), PP2Acat and PP2A (PR65) were stripped and reprobed with control anti-β-actin, anti-GAPDH or anti-γ-tubulin antibodies. Linearity of all antibodies was confirmed by a 2-fold dilution series of euploid and Tc1 cortical samples. Relative signal of antibody of interest compared with the internal control was then calculated, and relative signal was then normalized to mean relative signal of littermate sex-matched control samples.

Primary antibodies against DYRK1A (7D11, Abnova, Taipei City, Taiwan) 1/500, pantau (DAKO, Glostrup, Denmark) 1/1600, phospho-tau Ser396/404 (PHF1, kind gift of P. Davies) 1/100; phospho-tau Ser202/Thr205 (AT8, Thermo Scientific, Loughborough, UK) 1/100, phospho-tau Thr212 (Invitrogen) 1/100, PP1 (E-9, Santa Cruz, Santa Cruz, CA, USA) 1/1000, PP2A (PR65A) (kind gift of S.M. Dilworth) 1/250, PP2Acat (kind gift of S.M. Dilworth) 1/1000, pan-GSK-3β (BD Biosciences, Oxford, UK) 1/5000, phospho-GSK-3β Ser9 (Cell Signaling Technologies, Boston, MA, USA) 1/1000, phospho-GSK-3β Tyr216 (BD Biosciences) 1/1000, CDK5 (Millipore, Billerica, MA, USA) 1/2000, p35/p25 (Santa Cruz) 1/200, total-AKT (Cell Signaling Technologies) 1/100, phospho-AKT Ser473 (Cell Signaling Technologies) 1/250, β-actin (Sigma, St Louis, MO, USA) 1/80,000, Glyceraldehyde 3-phosphate dehydrogenase (Abcam, Cambridge, UK) 1/20,000, γ-tubulin (Sigma) 1/10,000, were used at the concentrations indicated. We note that linearity of antibody binding signal for all antibodies, was confirmed. A dilution series of Tc1 and euploid control cortical total proteins was used for this.

### 2.6 Immunohistochemistry

Whole brains of Tc1 and age- and sex-matched wild type littermate mouse were fixed by immersion in 10% buffered formal saline (Pioneer Research Chemicals Colchester, Essex, UK) for a minimum of 48 hours. Following further washing for 24 hours in 10% buffered formal saline, tissue samples were processed and embedded in paraffin wax. Sections were cut at a thickness of 5 μm. After dewaxing sections were pretreated by protease digestion. Staining with anti-tau antibodies was undertaken using a Ventana automated immunohistochemical staining machine (Ventana Medical Systems, Tuscon, AZ, USA) as described previously (Wadsworth et al., 2008). A biotinylated-anti-rabbit IgG secondary antibody (iView SA-HRP, Ventana Medical Systems) was used before development with 3′3 diaminobenzedine tetrachloride as the chromogen (iView DAB, Ventana Medical Systems). Hematoxylin was used as the counterstain.

### 2.7 Statistical analysis

Data were analyzed by analysis of variance (ANOVA), fixed factors; genotype of mouse (Tc1 vs. control), age of mouse (2 months vs. 20 months of age), and tissue sampled (hippocampus vs. cortex). No significant effect of tissue sampled was found in the analysis therefore a further ANOVA, fixed factors; genotype of mouse (Tc1 vs. control) and age of mouse (2 months vs. 20 months of age) was undertaken. For significant fixed factors (*p* < 0.05) a post hoc comparison (the Fisher's least significant difference test) was applied.

## 3 Results

### 3.1 Age-dependent increase in tau phosphorylation in Tc1 mice

**Fig. 1:**
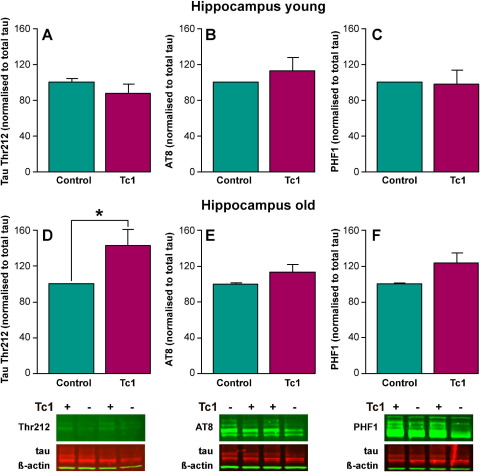
Tau is aberrantly phosphorylated at threonine 212 in the hippocampus of aged but not young Tc1 mice. The abundance of phosphorylated forms of tau was investigated by Western blot of total hippocampal protein lysates using anti-phospho-tau antibodies Thr212 (A) and (D), AT8 (B) and (E), and PHF1 (C) and (F). Equal amounts of total protein were loaded per lane and anti-total tau and β-actin antibodies were used as controls (A–F). Relative phospho-tau/total-tau signal was determine for each Tc1 sample and compared with the phospho-tau/total-tau ratio detected in the matched littermate nontranschromosomic control, in both young (2 months of age) and old (20 months of age) animals. Normalized signals were analyzed by analysis of variance (ANOVA) including the fixed factor of tissue type (hippocampus or cortex) and no significant effect of tissue was observed therefore data for both types of tissues was combined for subsequent analysis. A further ANOVA using fixed factors genotype of mouse (Tc1 or control) and age of mouse (2 months of age or 20 months of age) was performed. Tau Thr212 (normalized to total tau) signal was significantly affected by the interaction of genotype of mouse × age (*F*(1,51) = 6.110; *p* = 0.17). Post hoc tests showed that a significant increase in tau phosphorylated at Thr212 was detected in old Tc1 hippocampus compared with age- and sex-matched nontranschromosomic littermate control (least significant difference [LSD] *p* = 0.017; *n* = 8) and also old Tc1 hippocampus compared with young Tc1 hippocampus (LSD *p* = 0.04; *n* = 7). No significant increase in tau phosphorylated at Thr212 was detected in the hippocampus of young Tc1 mice compared with age- and sex-matched controls (2 months of age) (*n* = 7). No significant increase in phospho-tau detected by AT8 or PHF1 was detected in aged or young Tc1 hippocampus compared with wild type littermate control hippocampus (*n* = 9–10). Error bars show standard error of the mean.

To determine if trisomy of chromosome 21 alters the phosphorylation of tau, we assayed a number of tau sites that are aberrantly phosphorylated in AD. We studied 3 phospho-tau-specific antibodies in detail; PHF1 (Ser396/Ser404), AT8 (Ser202/Thr205), and Thr212 (Thr212); that recognize sites that are conserved in both mouse and human tau. Phospho-tau-specific signal was normalized against total tau signal. Young mice aged 2 months and old mice aged 20 months were studied as indicated. The total tau levels detected did not differ between the brains of Tc1 and control euploid mice (Supplementary Fig. S2). Low abundance of tau phosphorylated at sites recognized by PHF1, AT8, or Thr212 antibodies was detected in the hippocampus of young Tc1 and control mice, aged 2 months. No significant increase in tau phosphorylated at these sites was detected in the hippocampus of young Tc1 mice, compared with matched wild type littermate controls (Fig. 1A, B, C).

**Fig. 2:**
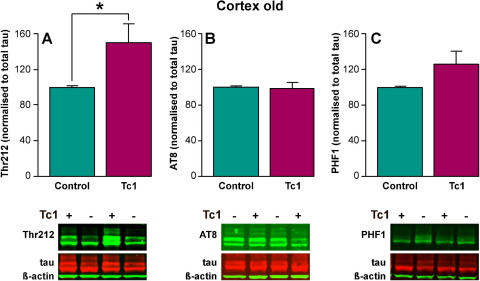
Tau is aberrantly phosphorylated at threonine 212 in the cortex of aged Tc1 mice. The abundance of phosphorylated forms of tau was investigated by Western blot of total cortical protein lysates using anti-phospho-tau antibodies Thr212 (A), AT8 (B), and PHF1 (C). Equal amounts of total protein were loaded per lane and anti-total tau and β-actin antibodies were used as controls (A–C). Relative phospho-tau/total-tau signal was determine for each Tc1 sample and compared with the phospho-tau/total-tau ratio detected in the matched littermate nontranschromosomic control. Post hoc tests showed that a significant increase in tau phosphorylated at Thr212 was detected in old Tc1 cortex (20 months of age) compared with matched wild type littermate control cortex (LSD *p* < 0.04; *n* = 10). No significant increase in phospho-tau detected by AT8 or PHF1 was detected in aged or young Tc1 cortex compared with euploid control cortex (*n* = 7–10). Error bars show standard error of the mean.

An increase in tau phosphorylated at Thr212 was observed in the hippocampus of old Tc1 mice compared with aged matched control mice and also young Tc1 mice, by Western blot (ANOVA genotype × age *F*(1,51) = 6.110; *p* < 0.017; post hoc least significant difference [LSD] test old Tc1 compared with old control *p* = 0.017, and young Tc1 compared with old Tc1 *p* = 0.04) (Fig. 1D). No significant increase in phosphorylation of tau recognized by PHF1 and AT8 was observed in old Tc1 hippocampus; although a trend to increased phosphorylation at the PHF1 site was observed (Fig. 1E and F). An increase in Thr212 signal was detected in the cortex of Tc1 mice aged 20 months compared with aged-matched control mice (post hoc LSD test *p* = 0.04) (Fig. 2). Thus in old Tc1 mice elevated phosphorylation of tau at Thr212 occurs in the brain. This suggests that trisomy of Hsa21 leads to the accumulation of aberrantly phosphorylated tau in the absence of any additional copies of APP.

These data indicate that in the aged Tc1 brain abnormal phosphorylation of tau occurs, which may promote the formation of NFT. To determine if NFT or other tau aggregates occur in the brains of aged Tc1 mice, sections of Tc1 brain were stained with Thr212 and PHF1 antibodies (Supplementary Fig. S3). No significant staining in the brain could be detected using PHF1 or anti-tau Thr212 antibodies in old Tc1 or wild type littermate control mice. A previous study has demonstrated that no NFT could be detected using anti-tau antibody AT8 (O'Doherty et al., 2005). These data suggest that the aberrant phosphorylation of tau in brains of old Tc1 mice does not trigger the formation of NFT or detectable aggregates of tau in brain.

### 3.2 The levels of the phosphatases, PP2A, and PP1, are not altered in the Tc1 mouse

To further understand the phosphorylation status of tau in the context of chromosome 21 trisomy we investigated the expression of a number of key regulators of tau phosphorylation. Phosphorylation of tau is a dynamic process and phosphate groups can be removed from the protein by the action of a number of phosphatases. In particular protein phosphatase 2A (PP2A) and protein phosphatase (PP1) have a major role in regulating the phosphorylation of tau (Goedert et al., 1992; Liu et al., 2005; Matsuo et al., 1994; Papasozomenos and Su, 1995). The expression of the PP2A catalytic subunit (PP2cat) is dramatically decreased in the brains of people who have DS and AD and may contribute to disease pathogenesis (Liang et al., 2008). We investigated the abundance of the PP2A catalytic subunit, PP2A scaffold subunit (PR65/A) and PP1 catalytic (PP1) subunit in the brains of Tc1 mice. The level of the phosphatase subunits, detected by Western blot, was the same in the hippocampus of young and old Tc1 and wild type littermate control mice (Supplementary Fig. S4, and Table S1). Similarly no difference in the amount of PP1, PP2Acat, or PR65/A was observed in the cortex of aged Tc1 mice compared with wild type littermate control animals (Supplementary Fig. S4). These data suggest that the Hsa21 gene or genes required to modify the expression of PP2Acat may not be functionally trisomic in the Tc1 mouse model.

### 3.3 The expression of DYRK1A is elevated in the hippocampus and cortex of Tc1 mice

**Fig. 3:**
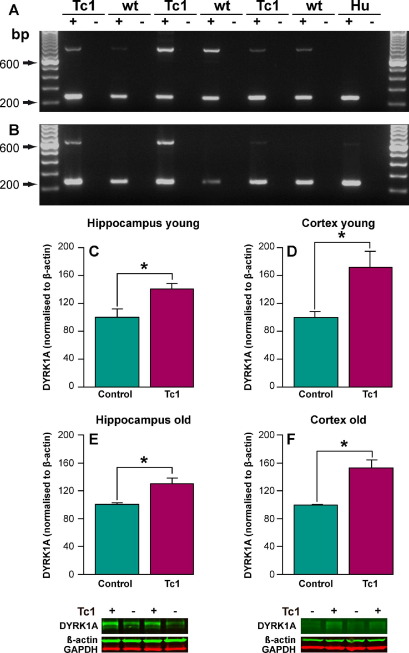
The expression of DYRK1A is elevated in the brains of Tc1 mice compared with wild type littermate control mice. The expression of mouse Dyrk1a and human DYRK1A transcript in Tc1 mouse brain was confirmed by RT-polymerase chain reaction (PCR) on total brain RNA from young Tc1 mice (2 months of age, *n* = 3). Primers that cross-react with both human and mouse Dyrk1A transcript product size 235 base pairs (A and B) were multiplexed with either specific for mouse *Dyrk1a* that produce a 793-base pair product (A) or primers specific for human *DYRK1A* that produce a 621-base pair product (B). The amount of DYRK1A protein in the hippocampus and cortex of Tc1 mice was quantified by Western blot of total protein lysates using anti-DYRK1A antibody 7D11, which is predicted to recognize both the human and mouse form of the protein (D–F). Equal amounts of total protein were loaded per lane and anti-β-actin and GAPDH antibodies were used as controls for total protein amount per lane (F) and (G). Relative DYRK1A/β-actin signal was determined for each Tc1 sample and compared with the signal for the matched wild type littermate control. An increase in DYRK1A was detected in the hippocampus and cortex of young (2 months of age) and old (20 months of age) Tc1 mice compared with wild type littermate control mice (analysis of variance [ANOVA] genotype *F*(1,47) = 723.076; *p* = 0.000; post hoc least significant difference [LSD] young hippocampus, *p* = 0.028, young cortex, *p* = 0.004, old hippocampus, *p* = 0.041, and old cortex, *p* = 0.000). Error bars show standard error of the mean.

Trisomy of the kinase *DYRK1A* has been previously linked to the hyperphosphorylation of tau in people with DS. The Tc1 mouse model of DS carries a copy of human *DYRK1A* in addition to the endogenous mouse *Dyrk1A* gene and thus the model is trisomic for this gene (Supplementary Fig. S5). Expression of mouse and human DYRK1A RNA transcripts can be detected in the brains of Tc1 adult mice (Fig. 3A and B). AD-related tau pathology is particularly prominent in the hippocampus and cortex and DYRK1A is expressed in both these regions in the adult mouse brain (Braak and Braak, 1991; Martí et al., 2003). To determine if elevated DYRK1A may contribute to the observed phosphorylation of tau in Tc1 mice, we investigated the abundance of this key protein in the hippocampus and cortex. An increase in DYRK1A signal, normalized to β-actin or GAPDH, was detected by Western blot in both the hippocampus and cortex of young adult and old Tc1 mice compared with wild type, age-, sex-, and litter-matched control animals (Fig. 3C–F, Supplementary Table S1) (ANOVA genotype *F*(1,47) = 723.076; *p* = 0.000, LSD post hoc young hippocampus *p* = 0.028, young cortex *p* = 0.004, old hippocampus *p* = 0.041, old cortex *p* = 0.000). This increase in protein level is consistent with trisomy of DYRK1A in the Tc1 mouse model and is similar to the increase in expression in the brains of adults with DS (Liu et al., 2008; Wegiel et al., 2008). Thus despite the clear elevation in DYRK1A expression in the Tc1 hippocampus in young mice, no elevation in tau phosphorylation is observed. We also observe no significant change in the amount of DYRK1A in young compared with old Tc1 mice or young compared with old control mice (Supplementary Fig. S6).

### 3.4 The abundance of CDK5 and its regulators is not altered in old Tc1 mice

In other mouse models of Hsa21 trisomy, Ts65Dn and Ts1Cje, and mouse models of DYRK1A overexpression, significant aberrant phosphorylation of tau at PHF1 and AT8 sites has been observed (Liu et al., 2008; Ryoo et al., 2007; Shukkur et al., 2006). Aberrant phosphorylation of tau is mediated by a number of kinases, including CDK5 (Noble et al., 2003; Plattner et al., 2006). Elevated abundance of CDK5 has been previously reported in adult hippocampus of an alternative mouse model of DS (Pollonini et al., 2008). Thus to understand the relatively restricted pattern of aberrant tau phosphorylation observed in an aged Tc1 model, we studied CDK5 level in the brains of old mice. We observed no significant alteration in the amount of CDK5 in the hippocampus or the cortex of old Tc1 mice, compared with wild type littermate control animals (Supplementary Fig. S7). The activity of CDK5 is regulated by binding to the neuronal specific cofactor p35/p25 (Tsai et al., 1994). p25 is a cleavage product of p35, it is more stable than its parent protein and hence a more potent activator of CDK5 activity (Patrick et al., 1999). Elevated p25/p35 ratios have been reported to occur in AD patients (Patrick et al., 1999; Tseng et al., 2002) and increased expression of p25 is linked with aberrant phosphorylation of tau (Noble et al., 2003; Plattner et al., 2006). We observed no increase in p35 signal in aged Tc1 cortex or hippocampus. We detected very low levels of p25 in all samples studied and observed no elevation of p25/p35 ratio in old Tc1 mice compared with controls (Supplementary Fig. S7). These data suggested that in old Tc1 mice the pattern of aberrant phosphorylation of tau is not mediated by a change in CDK5 level or activity.

### 3.5 GSK-3β is aberrantly phosphorylated in old but not young Tc1 mice

**Fig. 4:**
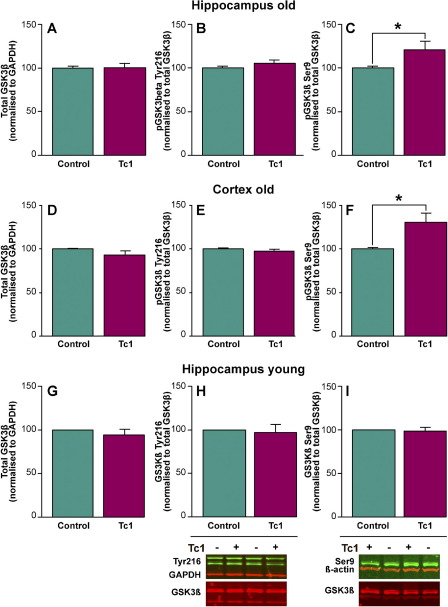
Phosphorylation of glycogen synthase kinase3-β (GSK-3β) at serine-9 is increased in the hippocampus and cortex of old Tc1 mice. The abundance of total GSK-3β and GSK-3β phosphorylated at Ser9 and Tyr216 was investigated by Western blot of total hippocampal (A–C) and (G–I) and cortical (D–F) protein lysates, from old (A–F) and young (G–I) Tc1 mice and matched wild type littermate control animals. Equal amounts of total protein were loaded per lane and anti-β-actin antibody signal was used as a control for amount of total protein loaded (A–I). Total GSK-3β signal was normalized to GAPDH signal in Tc1 samples, and compared with age- and sex-matched nontranschromosomic control signal. No significant difference in total GSK-3β in Tc1 samples was detected in old (20 months of age) hippocampus (*n* = 10) (A), old (20 months of age) cortex (*n* = 7) (D), or young (2 months of age) hippocampus (*n* = 7) (G). Phospho-GSK-3β signal was normalized to total GSK-3β signal in Tc1 samples, and compared with the respective matched littermate euploid control signal. No significant difference in GSK-3β phosphorylated at Tyr216 was detected in Tc1 compared with controls in old (20 months of age) hippocampus (*n* = 10) (B), old (20 months of age) cortex (*n* = 6) (E), or young (2 months of age) hippocampus (*n* = 6) (H). A significant increase GSK-3β phosphorylated at Ser9 was detected in old (20 months) Tc1 brain (analysis of variance [ANOVA] genotype × age *F*(1,45) = 5.482; *p* = 0.024) by post hoc least significant difference (LSD) tests significant increases in signal were detected in aged Tc1 hippocampus (*p* = 0.011) (C) and old (20 months of age) cortex (*p* = 0.003) (F) compared with age- and sex-matched control samples. No significant increase in phosphorylation of GSK-3β at Ser9 was detected in young Tc1 hippocampus (*n* = 7) (I). Error bars show standard error of the mean.

In vitro phosphorylation of tau by DYRK1A primes the protein for further phosphorylation by the kinase GSK-3β (Liu et al., 2008; Woods et al., 2001). Our data suggest that this process does not occur readily in old Tc1 mice. To investigate this further we determined the amount and phosphorylation status of GSK-3β in the aged Tc1 brain. No significant change in the level of this key kinase was observed in the brains of Tc1 mice (Fig. 4A, D, and G, and Supplementary Fig. S8). GSK-3β is constitutively active, and kinase activity has been correlated with phosphorylation of GSK-3β Tyr216 (Hughes et al., 1993). No difference in phosphorylation of GSK-3β at Tyr216 was observed in old Tc1 mice compared with wild type littermate control animals (Fig. 4B, E, and H). Activity of GSK-3β can be significantly inhibited by phosphorylation of N-terminal Ser9, which when phosphorylated prevents substrate access to the catalytic kinase site (Cohen and Frame, 2001; Sutherland et al., 1993). In the cortex and hippocampus of aged Tc1 mice we observed an increase in phosphorylation of GSK-3β at Ser9 compared with control animals (Fig. 4C and F) (ANOVA genotype × age *F*(1,45) = 5.482; *p* = 0.024, LSD post hoc hippocampus *p* = 0.011, cortex *p* = 0.003). This change in phosphorylation suggests that GSK-3β activity may be decreased in both the hippocampus and cortex of old Tc1. This may contribute to the relatively limited extent of aberrant tau phosphorylation observed in old Tc1 brains, as compared with other aged mouse models that overexpress DYRK1A. Interestingly, no increase in phosphorylation of GSK-3β Ser9 is observed in young adult Tc1 hippocampus (Fig. 4I). Therefore, the altered phosphorylation of GSK-3β caused by trisomy of Hsa21 in the Tc1 mouse model is an age-dependent phenotype.

### 3.6 AKT is aberrantly phosphorylated in old Tc1 mice

**Fig. 5:**
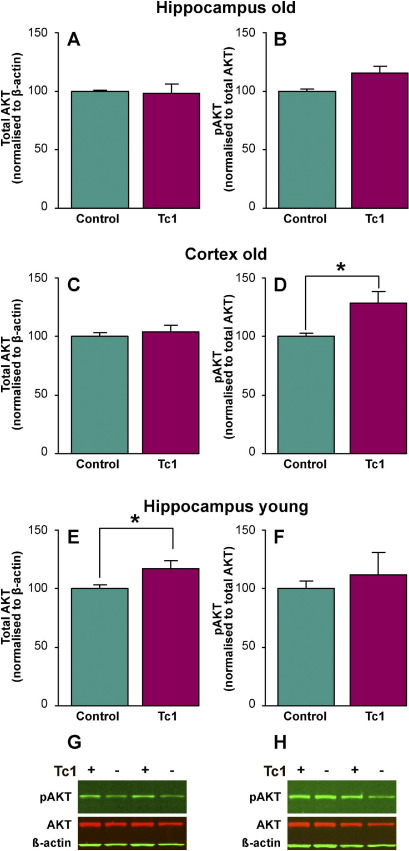
Phosphorylation of AKT at serine-473 is increased in the cortex of old Tc1 mice. The abundance of total AKT and AKT phosphorylated at Ser473 was investigated by Western blot of total hippocampal (A–B) and (E–G) and cortical (C–D) and (H) protein lysates, from young (2 months of age) (E) and (F) and old (20 months of age) (A–D) and (G–H) Tc1 mice and age- and sex-matched wild type littermate control animals. Equal amounts of total protein were loaded per lane and anti-β-actin antibody signal was used as a control for amount of total protein loaded (A–H). Total AKT signal was normalized to β-actin signal in Tc1 samples, and compared with the respective matched wild type littermate control signal. No significant difference in total AKT in Tc1 samples was detected in old (20 months of age) hippocampus (*n* = 12) (A) or cortex (*n* = 12) (C). A significant increase in total AKT signal was detected in young hippocampus (analysis of variance [ANOVA] genotype *F*(1,47) = 4.570; *p* = 0.038; least significant difference [LSD] post hoc *p* = 0.024) (E). Phospho-AKT signal was normalized to total AKT signal in Tc1 samples, and compared with the respective matched littermate control signal. A significant increase in AKT phosphorylated at Ser473 was detected in old (20 months) Tc1 cortex (ANOVA genotype *F*(1,54) = 4.975; *p* = 0.030; post hoc LSD *p* = 0.013) (D). Error bars show standard error of the mean.

GSK-3β Ser9 is not located at a consensus DYRK1A phosphorylation site, and there is no evidence to suggest that DYRK1A can directly phosphorylate GSK-3β (Himpel et al., 2000). Thus, the observed alteration of phosphorylation of GSK-3β Ser9 is likely to be mediated by other kinases. AKT is known to target the GSK-3β Ser9 site (Cross et al., 1995); activity of AKT is up-regulated by phosphosphorylation at Ser473 (Alessi et al., 1996) and an increase in phosphorylated AKT has been reported to occur in the Ts65Dn and Ts1Cje mouse models of Hsa21 trisomy (Siarey et al., 2006; Siddiqui et al., 2008). We investigated if phosphorylation of AKT at Ser473 is altered in the brain of old and young Tc1 mice, as this may contribute to the change in phosphorylation of GSK-3β observed. In the cortex an increase in phosphorylated AKT signal was observed in old Tc1 mice compared with age- and sex-matched controls (Fig. 5D) (ANOVA genotype *F*(1,54) = 4.975; *p* = 0.030; post hoc LSD old Tc1 cortex compared with age-matched sample, *p* = 0.013). This alteration may contribute to the observed change in phosphorylation of GSK-3β Ser9 in old Tc1 mice. No difference in the abundance of total AKT was observed in either the cortex or hippocampus of old Tc1 mice (Fig. 5A and C). However we did observe a significant increase in total AKT in young Tc1 hippocampus compared with control samples (ANOVA genotype *F*(1,47) = 4.570; *p* = 0.038; LSD post hoc, *p* = 0.024). In the hippocampus of young Tc1 mice no significant increase in AKT phosphorylated at Ser473, relative to total AKT, could be detected (Fig. 5E).

## 4 Discussion

People who have DS have an elevated risk of developing AD but the mechanism underlying this phenotype is not fully understood. Hyperphosphorylation of tau is associated with the development of AD in people with and without DS; here we study whether trisomy of chromosome 21 genes alters the abundance of key regulators of tau phosphorylation and the phosphorylation state of tau, in a unique trisomic model of DS that carries a copy of Hsa21. The data presented here suggests that trisomy of Hsa21 genes leads to aberrant phosphorylation of tau at one site (Thr212) in an age-dependent manner but that this does not trigger the formation of NFT in the Tc1 mouse model. Furthermore, we show Hsa21 trisomy also causes the aberrant phosphorylation of AKT and GSK-3β, which may lead to a decrease in GSK-3β activity in the brains of old trisomic mice, highlighting a novel pathway through which Hsa21 trisomy may interact with aging mechanisms. The Tc1 mouse model is not functionally trisomic for APP (personal communication, S. Gribble, Wellcome Trust, Sanger Institute, and Supplementary Fig. S1). Thus the aberrant phosphorylation observed occurs independently of an extra copy of this important AD-associated gene; notably overexpression of mutant forms of APP promotes tau hyperphosphorylation (Gotz et al., 2010). Our data suggest that the activity of DYRK1A and other kinases, including AKT and GSK-3β, maybe important to the phosphorylation status of tau in Hsa21 trisomic models.

The tau site aberrantly phosphorylated in the aged Tc1 mice has been previously shown to be targeted by the Hsa21 kinase DYRK1A (Liu et al., 2008; Ryoo et al., 2007; Woods et al., 2001). Here we show that expression of this kinase is upregulated in our mouse model, consistent with the change observed in the brains of people with DS (Dowjat et al., 2007; Liu et al., 2008; Lockstone et al., 2007; Wegiel et al., 2008). Previous studies have demonstrated a correlation between DYRK1A expression level and kinase activity (Liu et al., 2008), suggesting that increase DYRK1A kinase activity occurs in the brains of Tc1 mice. We found that the increase in DYRK1A protein was similar in young and aged animals, indicating that aging does not modify the effect of trisomy on DYRK1A expression in vivo. However, in young animals the increase in DYRK1A in the hippocampus is not correlated with an increase in aberrant tau phosphorylation suggesting the young trisomic brain may be protected from accumulating this potentially harmful form of tau. Many processes are known to be important to the formation of aberrantly phosphorylated tau; including the accumulation of amyloid, enhanced kinase activity, and decreased phosphatase activity (Gotz et al., 2010); in young mice these processes may be efficiently regulated so as to prevent the accumulation of aberrantly phosphorylated tau.

Aberrant phosphorylation of tau at Ser202 occurs in mice that overexpress DYRK1A (Ryoo et al., 2007). Although DYRK1A can phosphorylate tau at serine-202, in vitro, the efficiency of this reaction is low compared with that of Ser212 (Ryoo et al., 2007). In this study we do not observe a significant increase in tau phosphorylated at 202/205 as detected by AT8 in aged Tc1 brain. This may occur because the total level of DYRK1A in our trisomic model is insufficient to cause elevated phosphorylation at this site. Alternatively, factors other than DYRK1A may contribute to the phosphorylation of tau at this site, and may be differentially regulated in the Tc1 mice compared with other models.

Previous experiments have suggested that the increased activity of DYRK1A primes tau for further phosphorylation by GSK-3β at a number of sites (Woods et al., 2001). Here we show an increase in phosphorylation of GSK-3β at a site (Ser9) that inactivates the kinase's activity; this may contribute to the limited number of tau phosphorylation sites at which phosphorylation is altered in the Tc1 model. Alternative mouse models of DS (Ts65Dn and Ts1Cje) that are trisomic for less than 60% of Hsa21 genes exhibit increased phosphorylation of tau at a number of sites, including those that were not significantly changed in this study (Liu et al., 2008; Ryoo et al., 2007). Consistent with our results phosphorylation of GSK-3β Ser9 is not altered in young Ts1Cje mice that model some aspects of DS (Shukkur et al., 2006). Phosphorylation at this site has not been previously studied in aged mouse models of DS, but elevated phosphorylation of GSK-3β Ser9 occurs in the brains of aged people with DS (Swatton et al., 2004) and thus the Tc1 mouse provides a model to further investigate this important phenotype.

Aberrant phosphorylation of tau at Thr212 was observed in Tc1 brains from mice that were aged 20 months but no such change was observed in young mice. Aging is the single greatest risk factor for the development of AD (Evans et al., 1989); aberrantly phosphorylated tau has been reported in aged people who do not have AD and the extent of tau deposition correlates with age (Buee et al., 2000; Delacourte et al., 1999). Age-dependent aberrant phosphorylation of tau has also been reported in rodent models (Plattner et al., 2006). Numerous different mechanisms have been suggested to mediate the effect of aging on tau phosphorylation; including the influence of changes in insulin signaling on GSK-3β activity; the effect of deregulation of calcium signaling on CDK5 activity; the role of mitochondrial decline, and oxidative stress (Hernández and Avila, 2008; Mandelkow et al., 1992; Swerdlow and Khan, 2009; Yu et al., 2009). An increase in GSK-3β activity is observed in aged rats (Lee et al., 2006). The data presented here suggest that trisomy of Hsa21 may modulate the effect of aging on GSK-3β activity.

GSK-3β also has a role in neuronal development and survival and has been implicated in a number of neurological disorders (Beasley et al., 2001; Cowper-Smith et al., 2008; Hooper et al., 2008; Jope and Roh, 2006). Inhibitors of GSK-3β have been shown to help protect neurons against neurotoxic stimuli and an increase in GSK-3β expression reportedly increases apoptosis (Chin et al., 2005; Saito et al., 2010). The altered phosphorylation of GSK-3β observed in the Tc1 mice may be neuroprotective. There is much interest in whether GSK-3β inhibitors can be used as a therapeutic tool for AD, because of the link between GSK-3β activity and tau phosphorylation (Hooper et al., 2008; Muñoz-Montaño et al., 1997). However, there is no general consensus as to whether and how GSK-3β activity is altered in AD patients (Blalock et al., 2004; Hye et al., 2005; Leroy et al., 2007; Pei et al., 1997; Swatton et al., 2004). The data presented here suggest that models of trisomy of Hsa21 may provide insight into the regulation of GSK-3β activity. Inhibition of GSK-3β and aberrant phosphorylation of tau are also associated with defects in working and spatial memory (Arendash et al., 2004; Bell et al., 2005; Hu et al., 2009; Tan et al., 2010) and may contribute to learning deficits in aged Tc1 mice.

GSK-3β is a serine/threonine kinase and is a prominent member of the Wnt-β-catenin cell signaling pathway (Hur and Zhou, 2010; Wu and Pan, 2010). Inactivation of GSK-3β can be triggered by changes in cell survival or insulin signaling (reviewed by Hur and Zhou, 2010); these signaling pathways may be modified in DS. A number of different kinases can phosphorylate GSK-3β Ser9, including AKT, ribosomal protein S6 kinase, and S6 kinase (Cross et al., 1995; Kim and Kimmel, 2000; Sutherland et al., 1993). Here we show phosphorylation of AKT Ser473 is enhanced in the brains of old Tc1 mice. Phosphorylation of AKT activates this kinase and hence may contribute to the observed change in GSK-3β. Altered phosphorylation of AKT has also been observed in Ts65Dn and Ts1Cje mice, that model aspects of of DS (Siarey et al., 2006; Siddiqui et al., 2008).

The identity of the kinase that phosphorylates AKT at Ser473 is much debated, candidates included the mTOR protein kinase-rictor complex (Sarbassov et al., 2005). The region surrounding AKT Ser473 is not similar to the proposed DYRK1A consensus sequence (Himpel et al., 2000), and thus is unlikely to be a direct target of this kinase. Other Hsa21 trisomic genes may also contribute to the aberrant phosphorylation of AKT observed in the brains of old Tc1 mice; indeed overexpression of the Hsa21 encoded *SOD1* gene has been linked to increase phosphorylation of AKT at Ser473 in brain, in the context of stress (Endo et al., 2007; Noshita et al., 2003). Further investigation is required to determine the mechanism by which the phosphorylation AKT is modified in Hsa21 trisomy.

Recently, the expression of DYRK1A has been shown to exhibit circadian oscillations, with highest levels of expression occurring midway through the dark period (Kurabayashi et al., 2010). We note that all tissue samples presented in this report were collected between hours 1 and 6 of the light cycle, when DYRK1A is half its maximal level (Kurabayashi et al., 2010). Higher levels of DYRK1A could magnify or modify the effects observed here, such that more extensive phosphorylation of tau at Thr212 may occur at night.

## 5 Conclusions

The Tc1 mouse carries a freely segregating copy of Hsa21 and is a unique animal model of DS. Here we use this model to study the influence of Hsa21 trisomy on tau phosphorylation in both young adult and aged mice. We show that in the aged Tc1 brain tau is aberrantly phosphorylated at Thr212; this phosphorylation site is known to be a target of the Hsa21 kinase, DYRK1A. Consistent with this, Tc1 mice are trisomic for *DYRK1A* and overexpress the protein in brain throughout adult life. However, an increase in tau phosphorylated at Thr212 is not detected in young Tc1 animals. This novel result suggests that the young trisomic brain is protected from accumulating hyperphosphorylated tau despite the raised level of DYRK1A. Furthermore, in contrast to reports from alternative models of DS, aged Tc1 mice do not exhibit significantly increased phosphorylation of tau at sites other than Thr212. This may result from a decrease in GSK-3β activity in the aged Tc1 brain, which may counter further aberrant phosphorylation of tau after priming phosphorylation by DYRK1A. Alterations in the activity of key regulators of tau phosphorylation other than DYRK1A may also occur in people who have DS and could contribute to their risk of developing AD.

## Disclosure statement

The authors declare that they have no actual or potential conflicts of interest.

Mice were housed in controlled conditions in accordance with guidance issued by the Medical Research Council in Responsibility in the Use of Animals for Medical Research (1993) and all experiments were carried out under License from the UK Home Office and with Local Ethical Review panel approval.
