# Supplementary material for: Altered regulation of tau phosphorylation in a mouse model of down syndrome aging
Source: Neurobiol Aging. 2012 Apr;33(4-2):828.e31–44. doi: 10.1016/j.neurobiolaging.2011.06.025 (PMC3314962; doi:10.1016/j.neurobiolaging.2011.06.025)
Supplement: Supplementary Table 1 [file mmc1.doc]

**Supplementary Figure Legends**

**Supplementary Figure S1. Expression of APP in the Tc1 mouse brain is truncated**

Expression of human APP was analysed by RT-PCR from total brain RNA, from Tc1 and litter- and sex- matched control mice and total human brain RNA (Ambion). Primers specific for human APP exons 9 to 15 generated a product from both the human control RNA sample and Tc1 total RNA brain samples but not control mouse brain RNA. No product was observed in Tc1 brain RNA samples for RT-PCR reactions using primers for human APP exons 14 to 18. Thus although human APP transcript is expressed in the Tc1 brain it is not full length and thus the Tc1 mice are not functionally trisomic for APP.

**Supplementary Figure S2. Abundance to total tau in Tc1 brains is not significantly different from that of wild-type littermate control mice.**

The abundance of total tau was investigated by western blot of (A) total young hippocampal, (B) total old hippocampal and (C) total old cortical protein lysates from Tc1 mice and matched wild-type littermate control animals. Equal amounts of total protein were loaded per lane and anti-β-actin antibody signal was used as a control for amount of total protein loaded (A-C). Total GSK3β signal was normalized to β-actin signal in Tc1 samples, and compared with the respective matched littermate control signal. No significant difference in total tau in Tc1 samples was detected in (A) young (2 months of age) hippocampus (n = 8), (B) old (20 months of age) hippocampus (n = 10) or (C) old (20 months of age) cortex (n = 7). Error bars show standard error of the mean.

**Supplementary Figure S3. Neurofibrillary tangles and deposits of tau are not detected in aged Tc1 mice**

To investigate if aggregates of tau of neurofibrillary tangles occurred in the aged Tc1 brain sagital sections of Tc1 and wild-type littermate control brain were stained with phospho-tau specific antibodies Thr212 (A-C) and PHF1 (D-F). These antibodies detected neurofibrillary tangles and deposits of tau in sections of brain from an Alzheimer’s disease patient (A and D) but no significant signal was observed in sections of Tc1 brain from old mice (20 months of aged) (C and F) (n = 3). Photographs shown are frontal cortex. Paraffin embedded sections were pretreated with protease prior to incubation with primary antibodies, nuclei were counter-stained with haematoxylin. Scale bar = 50 μm.

**Supplementary Figure S4. Expression of PP1, PP2Acat and PR65/A in the young and old Tc1 hippocampus**

The abundance of PP1, PP2A(cat) and PP2A scaffold (PR65A) was investigated by western blot of total hippocampal protein lysates from old (A-C) and young (D-F) using antibodies against PP1 (A, D), PP2A(cat) (B, E) and PR65A (C, F). Equal amounts of total protein were loaded per lane and anti-β-actin or anti-GAPDH antibodies were used as controls for amount of total protein loaded per lane (A-F). The PP1, PP2A(cat) and PR65A signal was normalized to anti-β-actin or anti-GAPDH control signal for each Tc1 sample, and compared with the matched littermate euploid control. No significant difference in PP1, PP2A(cat) or PR65A abundance was detected in old Tc1 hippocampus (20 months) (n = 7-9). No significant difference in PP1, PP2A(cat) or PR65A abundance was detected in young Tc1 hippocampus (2 months) (n = 3). Error bars show standard error of the mean.

**Supplementary Figure S5. A copy of human DYRK1A is found in the Tc1 mouse**

Presence of a human DYRK1A gene in the Tc1 mice was confirmed by PCR of genomic Tc1 DNA extracted from tail tissue using two sets of human DYRK1A specific primers (product size 782 base pairs and 625 base pairs. Specificity of the primers was confirmed using a total human genomic DNA sample (HT) and wild-type littermate matched mouse genomic DNA sample (Tc1-) (n = 2). Identity of the PCR products generated was confirmed by sequencing and alignment with human reference sequence for DYRK1A.

**Supplementary Figure S6. A comparison of expression of DYRK1A in young and old Tc1 and control hippocampus.**

The abundance of DYRK1A was investigated by western blot of total hippocampal protein lysates. Equal amounts of total protein were loaded per lane and anti- β-actin antibody was used as controls for amount of total protein loaded per lane. The anti-DYRK1A signal was normalized to β-actin all samples, and the average signal observed in all young samples run per blot was then compared with the signal of hippocampus from old animals. Separate blots were run for control and Tc1 groups. No significant difference in DYRK1A signal was detected between young (2 months of age) and old (20 months of age of age) Tc1 or control samples (n = 8). Error bars show standard error of the mean. We note that because of the nature of this experiment, samples could not be matched by litter nor time of day of sample collection, these factors may contribute to the relatively high standard error observed.

**Supplementary Figure S7. Expression of CDK5, p35 and p25 in the old Tc1 hippocampus and cortex**

The abundance of CDK5, p35 and p25 was investigated by western blot of total cortical (A-C) and total hippocampal protein lysates (D-F). Equal amounts of total protein were loaded per lane and anti- β-actin or anti-GAPDH antibodies were used as controls for amount of total protein loaded per lane (A-F). The anti-CDK5 and anti-p35 signal were normalized to β-actin or anti-GAPDH control signal in Tc1 samples, and compared with the respective matched littermate wild-type control signal. The p25/p35 ratio in Tc1 samples was normalized to total β-actin signal and compared with the ratio observed in matched wild-type littermate control samples. No significant difference in CDK5, p35 abundance or p25/p35 ratio was detected in old Tc1 cortex or hippocampus (20 months of age of age) compared with matched controls (n = 4-9). Error bars show standard error of the mean.

**Supplementary Figure S8. A comparison of GSK3β and GSK3β phosphorylated at Ser9 signal in the young and old Tc1 and control hippocampus.**

The abundance of GSK3β and GSK3β phosphorylated at Ser9 was investigated by western blot of total hippocampal protein lysates. Equal amounts of total protein were loaded per lane and anti- GAPDH antibody was used as control for amount of total protein loaded per lane. The anti-GSK3β signal was normalized to GAPDH signal for all samples and the GSK3β Ser-9 signal was normalized to GSK3β signal. Then the average signal observed in all young samples run per blot was then compared with the signal of hippocampus from old animals run on that blot. Separate blots were run for control and Tc1 groups. No significant difference in GSK3β or GSK3β Ser9 signal was detected between young (2 months of age) and old (20 months of age of age) Tc1 or control samples (n = 8). Error bars show standard error of the mean. We note that because of the nature of this experiment samples could not be matched by litter this may contribute to the relatively high standard error observed.

Supplementary Table 1

| **Protein and tissue** | **Tc1 signal as percentage of wild-type littermate control normalized to GAPDH (+/- SEM)** |
| --- | --- |
| DYRK1A hippocampus (2 months of age) | 151.97 +/- 6.41 (p < 0.001 n = 7) |
| DYRK1A cortex (2 months of age) | 165.1 +/- 21.33 (p < 0.05 n = 4) |
| DYRK1A hippocampus (20 months of age) | 119.1 +/- 6.94 (p < 0.05 n = 8) |
| DYRK1A cortex (20 months of age) | 168.37 +/- 20.7 (p < 0.05 n = 7) |
| PP1 hippocampus (20 months of age) | 96.31 +/- 5.78 (ns n = 9) |
| PP2A(cat) hippocampus (20 months of age) | 97.14 +/- 9.52 (ns n =9) |
