# Supplementary material for: Altered regulation of tau phosphorylation in a mouse model of down syndrome aging
Source: Neurobiol Aging. 2012 Apr;33(4-2):828.e31–44. doi: 10.1016/j.neurobiolaging.2011.06.025 (PMC3314962; doi:10.1016/j.neurobiolaging.2011.06.025)
Supplement: Supplementary Figures 1 to 8 [file mmc2.pdf]

Figure S1

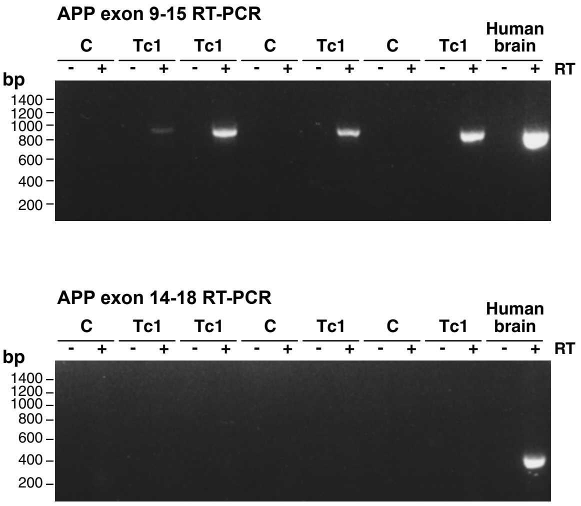
APP exon 9-15 RT-PCR
C
Tc1
Tc1
C
Tc1
C
Tc1
Human brain
- + - + - + - + - + - + - + - +
RT
bp
1400
1200
1000
800
600
400
200
APP exon 14-18 RT-PCR
C
Tc1
Tc1
C
Tc1
C
Tc1
Human brain
- + - + - + - + - + - + - + - +
RT
bp
1400
1200
1000
800
600
400
200

Figure S2

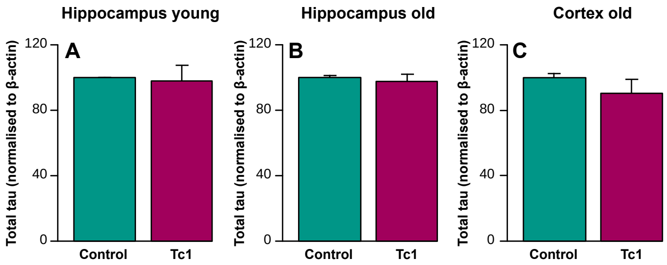

Hippocampus young
Hippocampus old
Cortex old
A
B
C
Total tau (normalised to β-actin)
120
80
40
0
Control
Tc1

Figure S3

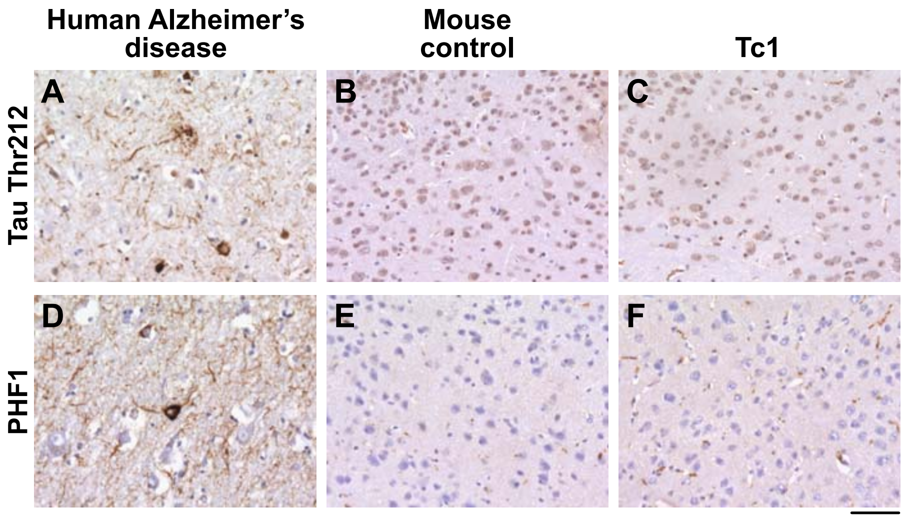

Human Alzheimer's disease
Mouse control
Tc1
Tau Thr212
PHF1
A
B
C
D
E
F

Figure S4

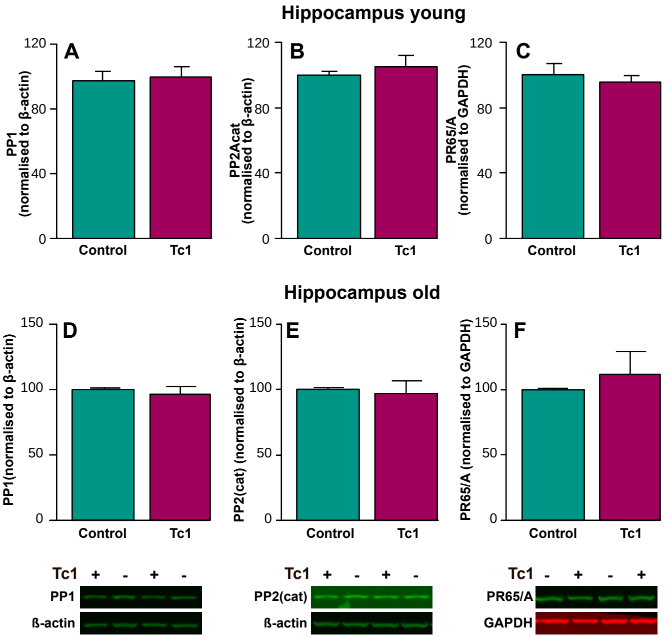
Hippocampus young
A
B
C
PP1 (normalised to β-actin)
PP2Acat (normalised to β-actin)
PR65/A (normalised to GAPDH)
120
80
40
0
Control
Tc1
Hippocampus old
D
E
F
PP1(normalised to β-actin)
PP2(cat) (normalised to β-actin)
PR65/A (normalised to GAPDH)
150
100
50
0
Tc1 + - + -
PP1
ß-actin
Tc1 + - + -
PP2(cat)
ß-actin
Tc1 - + - +
PR65/A
GAPDH

Figure S5

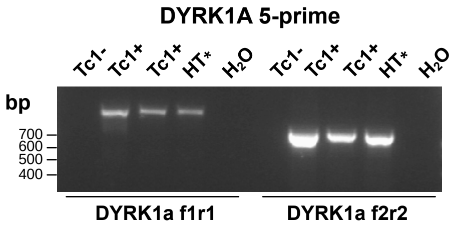

HT* = HT1080-neo targeted cell line used to make the Tc1 mice
Tc1- = littermates of Tc1+ mice that do not carry Hsa21

Forward primer	Sequence	Binding location	Reverse primer	Sequence	Binding location	Amplicon size
Dyrkf1	ATCCTCCTCGGGAAGAAGCC	21:37661451	Dyrkr1	GTGCATTGTCCTTGCGAATC	21:37662232	782
Dyrkf2	AGCCGAGGAGAGACTGAGCAG	21:37661631	Dyrkr2	AGCCGGCCCCATTTTCTTAAC	21:37662255	625

PCR product sequence confirmed (n = 2)

Figure S6

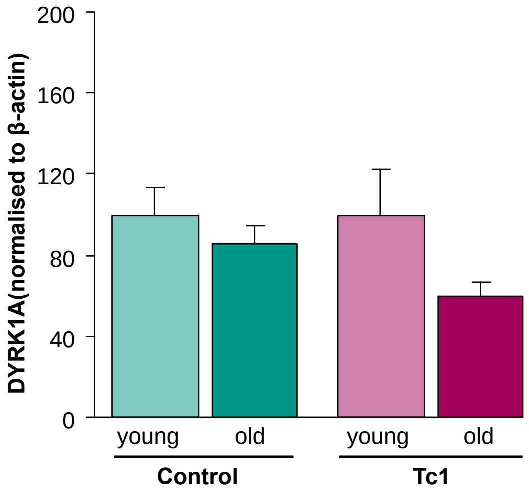

DYRK1A(normalised to β-actin)
200
160
120
80
40
0
young
old
young
old
Control
Tc1

Figure S7

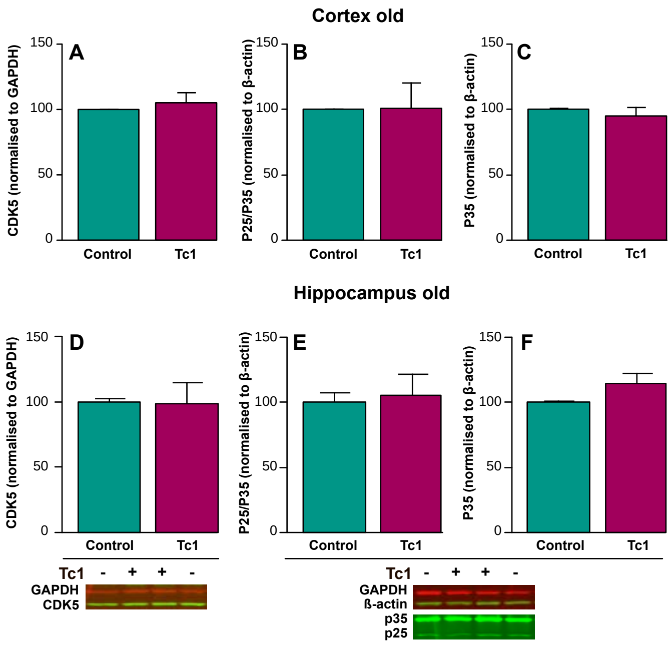

Cortex old
A
B
C
CDK5 (normalised to GAPDH)
P25/P35 (normalised to β-actin)
P35 (normalised to β-actin)
150
100
50
0
Control
Tc1
Hippocampus old
D
E
F
Tc1 - + + -
GAPDH
CDK5
GAPDH
ß-actin
p35
p25

Figure S8

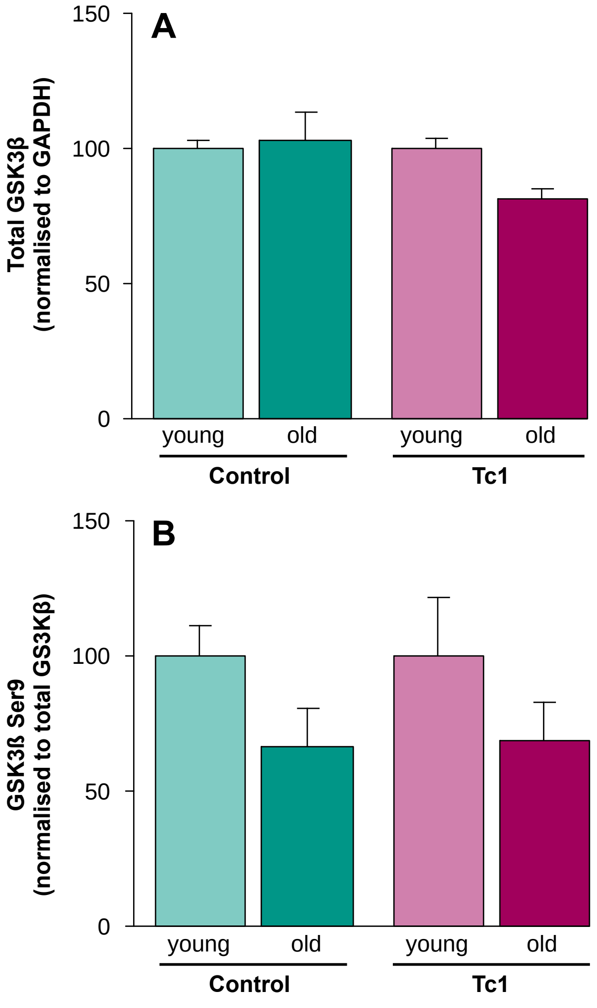

A
150
100
50
0
Total GSK3β (normalised to GAPDH)
young
old
young
old
Control
Tc1
B
150
100
50
0
GSK3ß Ser9 (normalised to total GS3Kβ)
young
old
young
old
Control
Tc1
